# The Outrigger Technique for Medial Opening Wedge High Tibial Osteotomy Using a Hintermann Distractor to Control the Osteotomy Gap Opening and the Posterior Tibial Slope

**DOI:** 10.1002/atn2.70032

**Published:** 2026-05-21

**Authors:** Sam Yasen, Ahmed Mabrouk

**Affiliations:** ^1^ Department of Trauma and Orthopaedics Basingstoke and North Hampshire Hospital Basingstoke England, U.K.

## Abstract

Medial opening wedge high tibial osteotomy is a successful procedure for correction of varus malalignment in isolation or in combination with other knee ligamentous and cartilage procedures. This article describes a technique for medial opening wedge high tibial osteotomy using a Hintermann distractor, which allows better control of the osteotomy gap opening and the posterior tibial slope. Preoperative digital planning is undertaken. A small longitudinal incision and a minimally invasive approach are performed to expose the proximal tibia. The Hintermann distractor is applied over 2 guide wires inserted divergently proximal and distal to the transverse osteotomy. The Hintermann distractor is used to open the osteotomy gap and maintain or change the posterior tibial slope according to the preoperative plan. Unimpeded access is provided, with no instrumentation in the gap, and this allows precise positioning of an allograft bone wedge, which is followed by fixation with an angular stable locking plate. In this technique, the Hintermann distractor serves as an outrigger that provides support to the osteotomy and offers rotational control to change the posterior tibial slope if desired.

## Graphical abstract

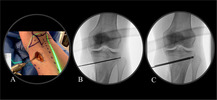

**VIDEO 1:** Patient is positioned supine. The left knee is shown. A video showing the surgical technique of medial opening wedge high tibial osteotomy using the Hintermann Distractor, highlighting its advantages in providing precise control of the osteotomy gap opening and maintaining optimal posterior tibial slope. Video content can be viewed at https://doi.org/10.1002/atn2.70032.

Medial opening wedge high tibial osteotomy (MOW HTO) has been proven as a highly rewarding procedure for cases with symptomatic isolated medial osteoarthritis (OA) and varus malalignment.^1^, ^2^ Traditional techniques, although effective in their outcomes, are prone to correction errors, which are more commonly those of undercorrection.^3^, ^4^, ^5^ Additionally, often reported are inadvertent changes in the posterior tibial slope (PTS), with a tendency for the slope to be increased following MOW HTO.^6^, ^7^ However, several modalities are being proposed to better improve correction accuracy.^8^, ^9^, ^10^ Moreover, there is an increased awareness of how to maintain the PTS in MOW HTO based on evidence from biomechanical and clinical studies.^11^, ^12^ Nevertheless, new techniques could serve a valuable addition to the armamentarium of a procedure that has witnessed substantial progression in the last decade.^13^, ^14^ The purpose of the described technique is to illustrate how to simultaneously control the osteotomy opening gap and the PTS using a Hintermann distractor, as an outrigger, through a minimally invasive approach.

## SURGICAL TECHNIQUE

### Indications and Contraindications

Patients are considered for surgery with moderate to severe knee pain; varus knee malalignment; and isolated medial compartment OA, after failure of conservative measures. There is no cutoff for age, body mass index, or the medial compartment OA grade. The procedure is contraindicated in cases with concomitant symptomatic patellofemoral OA or advanced grades of OA (Kellgren and Lawrence grade III or IV) in the lateral tibiofemoral compartment.

### Deformity Analysis and Preoperative Planning

Deformity analysis and preoperative planning are conducted digitally, on long leg standing radiographs (LSRs). MOW HTO is only undertaken in varus knees where the deformity analysis identifies this as being located in the tibia. The correction planning is bespoke for each patient according to the indication, anatomy, and pathology. Correction targets to restore the weight‐bearing axis to a Mikulicz point (weight‐bearing axis) of 50% to 58% are used depending on the OA grade of the medial compartment. Targets closer to 50% are planned for a more modest disease, and toward 58% for end‐stage arthritis with eburnation of bone. All key alignment indices are recorded. The planned correction angle and resultant opening wedge base are then derived from the calibrated digitally acquired long leg standing radiographs.

## SURGICAL PROCEDURE

The surgical procedure is shown in Video 1.

Patients are positioned supine and a thigh tourniquet is applied, which is inflated throughout surgery. The knee is flexed to 90° during the surgical approach, and extended during execution and opening of the osteotomy.

**FIGURE 1:**
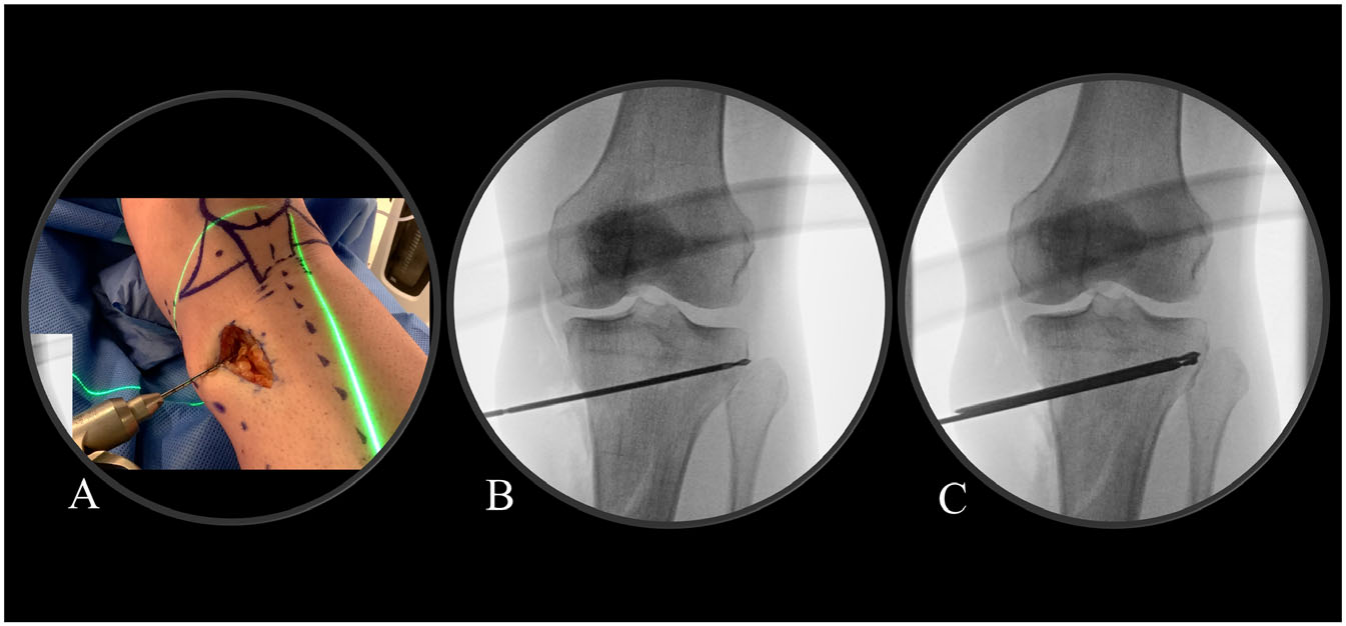
(A) Anteromedial view (cranial, toward the knee, at the top of the image; caudal, toward the ankle, at the bottom) of the left knee demonstrating the knee surface markings and the approach for the MOW HTO with insertion of the first Kirschner‐wire posteromedial. Intraoperative fluoroscopic image showing anteroposterior view of the proximal tibia. (B) Two 2.4 mm bendable osteotomy guide wires (Arthrex, Naples, FL) are inserted from the metaphyseal flare of the medial proximal tibia, and are driven toward hinge point. (C) The guide wires are bent away or snapped off (as in this image) and the saw blade can be seen in the main transverse plane of the osteotomy cut.

**FIGURE 2:**
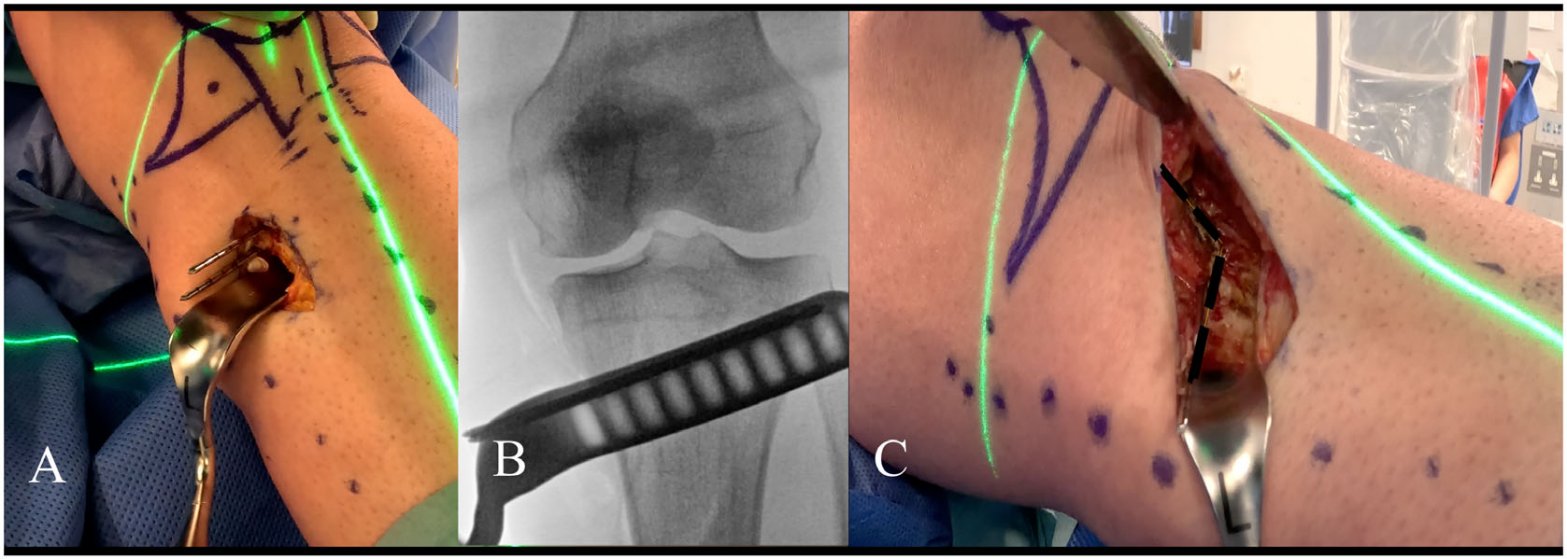
(A) Anteromedial view of the left knee (cranial, toward the knee, at the top of the image; caudal, toward the ankle, at the bottom) demonstrating the 2 K‐wires after being snapped off with a retractor protecting the posterior structures of the knee before performing the osteotomy. (B) Anteroposterior intraoperative fluoroscopic image demonstrating the 2 snapped off K‐wires and the semiradiolucent retractor, protecting the posterior knee structures while allowing visualization of the sawblade during the osteotomy. (C) Anteromedial view of the left knee (cranial, toward the knee, at the left of the image; caudal, toward the ankle, at the right of the image) demonstrating the 2 limbs of the biplanar osteotomy (black dashed lines) after completion and removal of the wires. (K‐wires, Kirschner wires.)

Under fluoroscopic guidance, two 2.4 mm bendable osteotomy guide wires (Arthrex, Naples, FL) are inserted from the metaphyseal flare of the medial proximal tibia, and are driven toward the proximal tip of the proximal tibiofibular joint. The wires are then bent away from the plane of the osteotomy for improved visualization. The transverse limb of the osteotomy is executed to within 5 mm of the lateral cortex using a precision saw blade (Stryker, Kalamazoo, MI) leaving sufficient space anteriorly for a biplanar cut Figure 1. A proximally orientated retro‐tubercular cut is then created posterior to the tibial tuberosity to subtend an angle of approximately 110° Figure 2.

**FIGURE 3:**
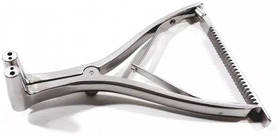
The Hintermann distractor with both its arms demonstrating it can accommodate 2 different sizes of wire.

**FIGURE 4:**
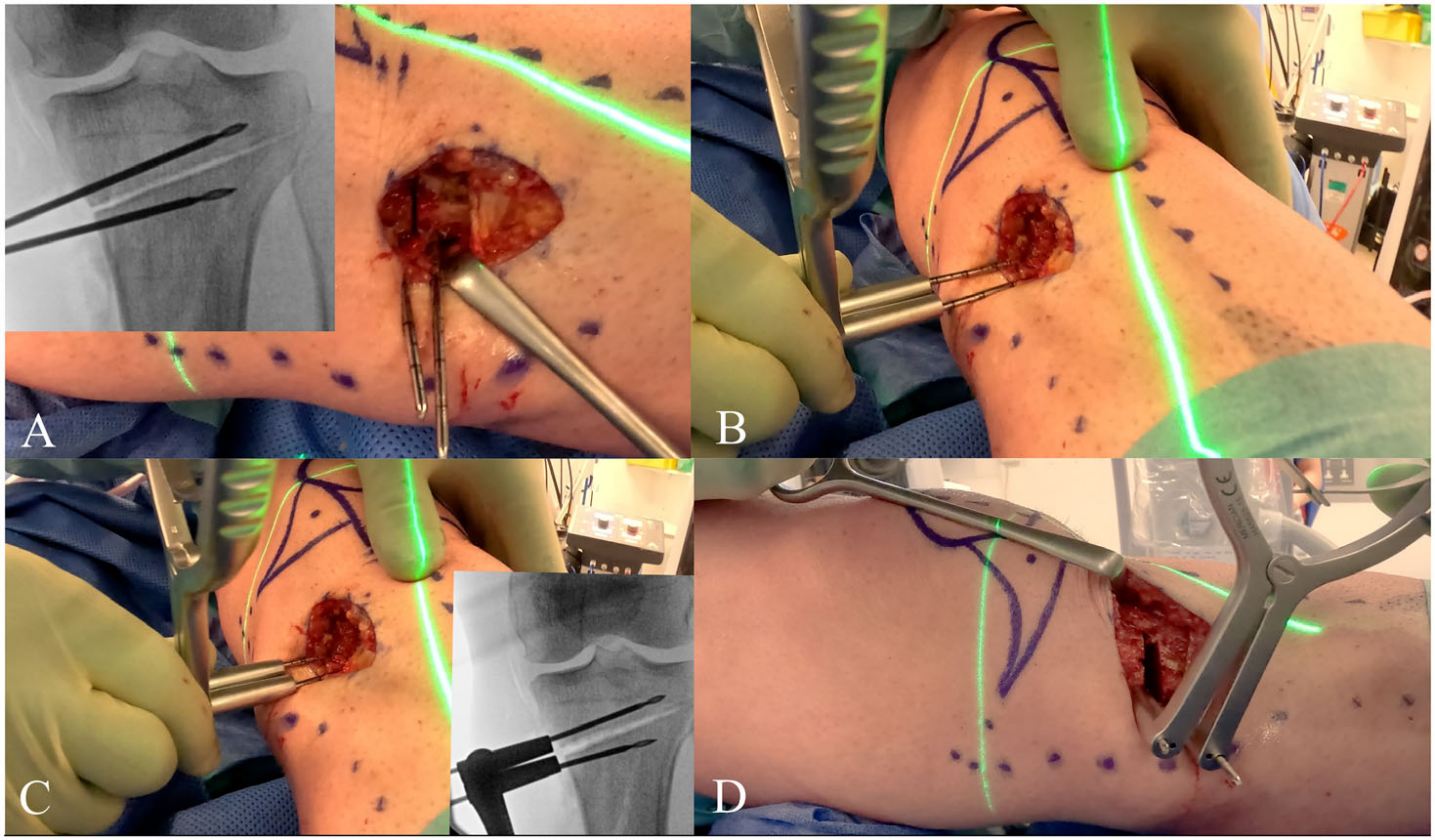
Intraoperative anteromedial images of the proximal tibia and fluoroscopic image showing anteroposterior view of the proximal tibia. A and D (cranial, toward the knee, at the left of the image; caudal, toward the ankle, at the right of the image) B and C (cranial, toward the knee, at the top of the image; caudal, toward the ankle, at the bottom). (A) Two snapped guidewires reinserted on either side of the transverse osteotomy divergently. The entry points for the 2 wires are directly adjacent to each other at the posterior edge of the medial proximal tibia, approximately 5 mm either side of the osteotomy. (B,C) The Hintermann distractor is slid over the wires down to the tibial cortex. (D) The Hintermann distractor is opened to the planned correction and the osteotomy opening is maintained while ensuring perfect sliding of the biplanar cut, avoid alteration of posterior tibial slope.

The guide wires are then snapped off, retrieved and reinserted either side of the transverse osteotomy. The entry points for the 2 wires are directly adjacent to each other, located at the posterior edge of the medial proximal tibia, approximately 5 mm either side of the osteotomy. The wires are driven in divergently toward, but not through, the contralateral cortices. The degree of divergence approximates the angular correction of the opening wedge, such that once the osteotomy is opened to the correct angle, the wires become roughly parallel. The wires are only inserted unicortical to avoid the risk of propagating an ascending or descending lateral hinge fracture. The Hintermann distractor (Figure 3) is slid over the wires down to the tibial cortex and opened progressively to the desired correction Figure 4.

Manual rotation of the retractor can directly influence the tibial slope at this stage. Maintaining the PTS is achieved by watching the biplanar aspect of the osteotomy sliding smoothly, rather than tilting. Corroboratory lateral fluoroscopic images can be taken if required to assess the PTS (but are typically not required). The anterior and posterior osteotomy gap opening distance is verified by direct measurement using calibrated calipers with an adjustment made to account for bone loss due to the swarf from the thickness of the saw blade. The Stryker precision blade is 1.27 mm thick, and therefore 1.3 mm is routinely added to the target for the planned correction.

**FIGURE 5:**
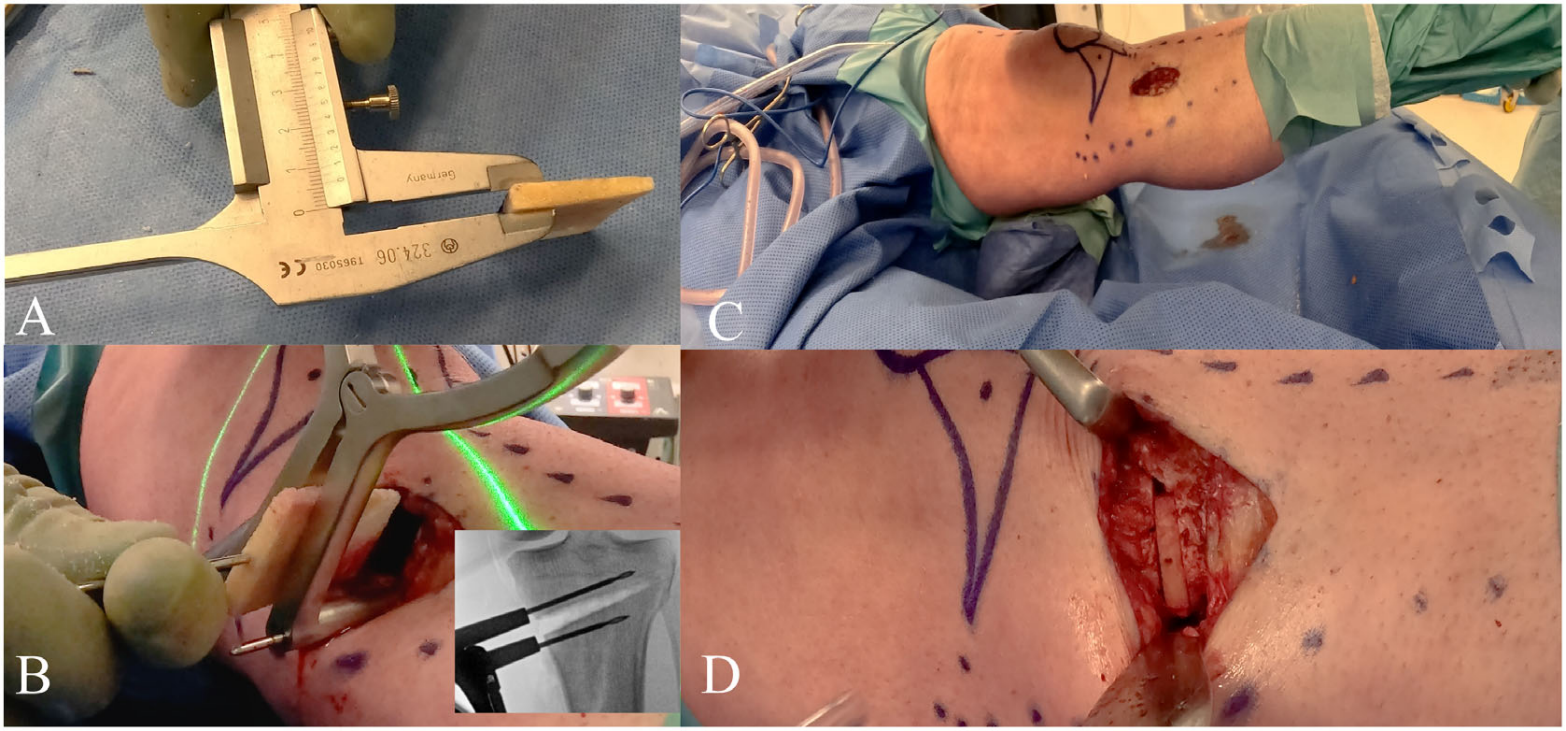
(A) Femoral head allograft fashioned in the form of a triangular bone wedge, the base of the triangle represents the planned correction and saw blade bone swarf. (B) Insertion of the femoral head allograft bone wedge through the opening between the arms of the Hintermann distractor using a Kirschner‐wire as a “joy‐stick” for insertion of the wedge. (C) The leg is lifted straight up with the correction maintained with the bone wedge only before fixation demonstrating stability. (D) Close view on the osteotomy gap filled up with the allograft bone wedge, positioned toward the posterior aspect of the transverse limb. The biplane limb can be seen to be well opposed. B and D (cranial, toward the knee, at the left of the image; caudal, toward the ankle, at the right of the image).

**FIGURE 6:**
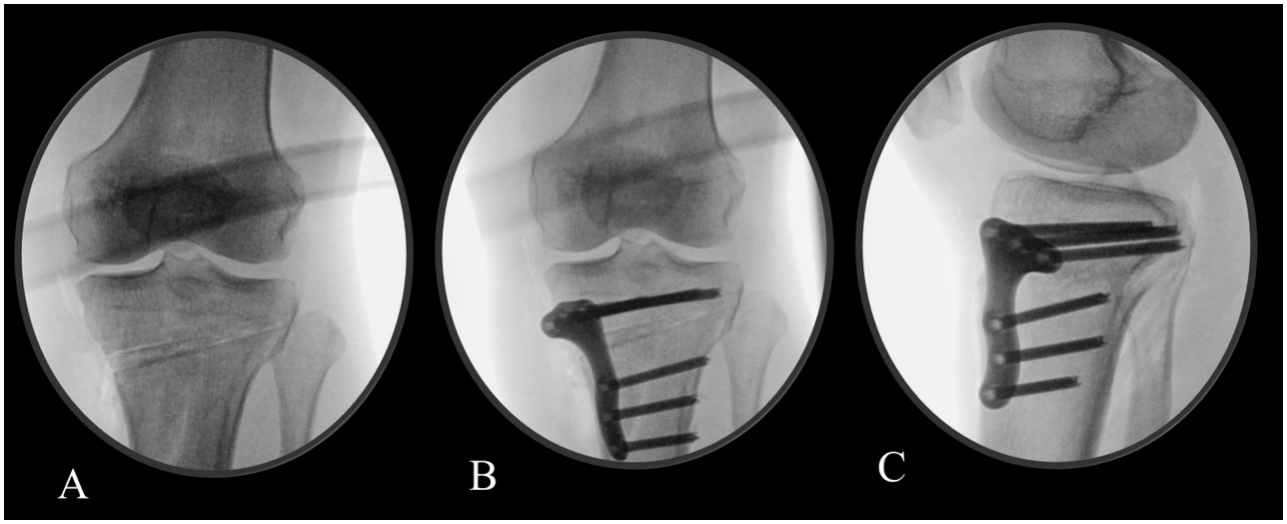
Intraoperative fluoroscopic image showing the proximal tibia. (A) Anteroposterior view showing the osteotomy with the correction completed and a fresh frozen bone allograft wedge inserted before plate fixation. (B) Anteroposterior view showing the medial opening wedge high tibial osteotomy completed and fixed with Newclip plate (Newclip Technics, Nantes, France). (C) Lateral view showing the osteotomy fixed and the posterior tibial slope maintained.

A fresh frozen femoral head allograft is custom‐fashioned into a trapezoidal shape with reduced thickness anteriorly compared with posteriorly and is inserted into the osteotomy gap. This is possible in an unrestricted manner as there is no instrumentation present withing the osteotomy gap. Once the wedge has been inserted, the Hintermann distractor and the guide wires are removed Figure 5. A well‐executed osteotomy is reasonably stable at this stage even prior to plate fixation. Osteotomy fixation is completed with an angular stable fixation plate Figure 6.

### Postoperative Protocol

Patients are allowed to immediately weight bear as tolerated (i.e., up to full weight bearing). Elbow crutches are used for protection within a physiotherapist‐supervised rehabilitation program, and typically discarded between 6 and 12 weeks. No brace is necessary. Early full range of motion is encouraged. Chemical thromboprophylaxis is given routinely using aspirin 75 mg daily for 6 weeks. Higher‐risk cases for venous thromboembolism are managed on case‐by‐case basis.

## DISCUSSION

MOW HTO has been reported to have high 5 and 10‐year survivorship of up to 97.2% and 91.9% respectively.^1^ Even HTO conversion to total knee arthroplasty (TKA) has been reported to have higher survival than TKA conversion following unicompartmental knee arthroplasty.^15^ However, for this procedure to preserve the knee effectively, high correction accuracy should be achieved along with avoidance of inadvertent changes in other alignment parameters. The presented technique offers better control of the osteotomy gap opening and the PTS with a readily available instrument; the Hintermann distractor. Pearls and pitfalls of its use are summarized in Table 1. In heavy machinery, an outrigger is an extendable support arm used to stabilize equipment and is crucial for maintaining balance and preventing tipping, especially when the machinery is lifting heavy loads or operating on uneven terrain. We have proposed the name for this approach as the “outrigger technique” due to similar role of the Hintermann distractor.

**TABLE 1:** Pearls and Pitfalls

**Pearls**	**Pitfalls**
1. The 2 K‐wires for Hintermann distractor have to be inserted a few mm staggered in depth at entry to facilitate smooth application of the Hintermann arms over the K‐wires	1. Bicortical insertion of the wires for Hintermann distractor increases the risk of Takeuchi type II or III lateral hinge fracture
2. Controlling the posterior tibial slope is performed by manual handling and rotation of the Hintermann distractor	2. Initial parallel wire insertion will result in wire conversion during osteotomy opening which can not be accommodated by the Hintermann retractor as it is parallel device
3. Inserting the wires in a divergent manner at approximately the same angle of the target correction ensures that the wires become approximately parallel when the osteotomy is opened to the correct degree. Mild additional divergence is safe and will confer a compressive force at the lateral hinge as the osteotomy is opened	3. If the wires are not inline in the sagittal plane upon entry, this will result in inadvertent translation or rotation of the osteotomy during opening

K‐wires, Kirschner wires.

Noyes et al.^16^ described the 3 triangle method to follow in MOW HTO to either maintain or change the PTS. However, their description was based on uniplanar MOW HTO. In the modern era, where the majority of osteotomies are performed in a biplanar manner, application of Noyes et al.^16^ principles could be challenged. The biplane osteotomy changes the algorithm of how much anterior opening should be performed compared with posterior opening. Due to the relatively triangular axial shape of the proximal tibia, the medial face of the tibia is inclined such that moving anteriorly also involves moving toward the midline, and thus moving closer to the lateral hinge point when viewed in the frontal plane. Hence the anterior opening gap measurement in any MOW HTO should be smaller than the posterior opening gap to maintain the PTS. Introducing a biplanar retro‐tubercular cut pushes the anterior extent of the main transverse osteotomy posteriorly and hence medially—and therefore further from the lateral hinge when viewed in the frontal plane. The thicker the biplane osteotomy, the further the anterior aspect that the main osteotomy cut is from the lateral hinge point, and the smaller the differential there needs to be between the posterior and anterior opening gap measurements. Maintaining the tibial slope can be achieved by ensuring that the biplanar cut opens in a sliding manner rather than with tilting occurring. This is more easily controlled and visualized with a Hintermann distractor applied, and can be additionally manually controlled.

The traditional technique involves using a Laminar spreader at the posterior aspect of the osteotomy, which results in opening with 2 fixed points and no control over the PTS. This results in a more challenging environment to control the PTS compared with the manual rotational control offered by using the Hintermann distractor in the presented technique. Additionally, the laminar spreader confers 2 further disadvantages first it obstructs the opening gap, which hinders placement of any allograft bone wedge, and secondly, while opening, if the tibial cortex is soft and becomes slightly compressed under the opening tension, this results in some loss of correction between the apparent measured gap and the true angular correction achieved. As the Hintermann distractor is used as an outrigger placed independently through the tibial cortex, not at the site of the osteotomy, this leaves it possible to make accurate measurements of the opening gap between the proximal and distal surfaces of the osteotomy.

The outrigger technique for MOW HTO using a Hintermann distractor over 2 guide wires placed either side of the main transverse osteotomy, as presented in this Technical Note, provides a simple, reproducible, safe and cost‐effective technique that offers better control of the planned correction in both the coronal and sagittal planes. The advantages and disadvantages of the outrigger technique is presented in Table 2.

**TABLE 2:** Advantages and Disadvantages of the Outrigger Technique for Medial Opening Wedge High Tibial Osteotomy

Advantages	
Control of the posterior tibial slope (PTS)	Medial opening wedge HTO is typically associated with an inadvertent increase in posterior tibial slope (PTS). The outrigger technique helps focus the surgeon's attention on tibial slope control, enabling straightforward maintenance of the preoperative tibial slope, or deliberate adjustment when dictated by the surgical plan.
Maintenance of the osteotomy gap	The Hintermann distractor is applied as an outrigger over wires inserted through the cortical bone. This avoids the risk of compressing the bone on either side of the osteotomy, as can occur with a laminar spreader or similar device, which may lead to underestimation and inaccurate measurement of the opening gap.
Access to osteotomy site	The outrigger technique provides unimpeded access to the osteotomy gap, facilitating straightforward insertion of a bone graft wedge.

Disadvantages	
Hinge fracture	Bicortical wire insertion for the Hintermann distractor, particularly distal to the osteotomy site, may increase the risk of propagating a type II hinge fracture.
Wire placement	The Hintermann distractor has parallel arms. Therefore, the wires should be inserted initially with slight divergence so that they become parallel as the osteotomy is opened. Failure to do so may prevent the distractor from opening the osteotomy successfully.
Hardware conflict	Using an oversized Hintermann distractor can obstruct fluoroscopic imaging and compromise image acquisition.

## DISCLOSURES

The authors (S.Y., A.M.) declare that they have no known competing financial interests or personal relationships that could have appeared to influence the work reported in this paper.
